# Transcatheter mitral valve replacement in native mitral valve with severe mitral annular calcification: Skirting the Sapien 3 to reduce the risk of paravalvular leaks

**DOI:** 10.3389/fcvm.2022.967473

**Published:** 2022-10-20

**Authors:** Alberto Pozzoli, Tiziano Torre, Giovanni Pedrazzini, Stefanos Demertzis, Enrico Ferrari

**Affiliations:** ^1^Division of Cardiac Surgery, Cardiocentro Ticino Institute, EOC Lugano, Lugano, Switzerland; ^2^Division of Cardiology, Cardiocentro Ticino Institute, EOC Lugano, Lugano, Switzerland; ^3^Faculty of Biomedical Sciences, Università della Svizzera Italiana (USI), Lugano, Switzerland

**Keywords:** Sapien 3 THV, MAC, heart surgery, mitral valve (ring calcification), mitral valve, mitral valve replacement, interventional cardiology

## Abstract

**Background:**

Mitral annular calcification (MAC) may represent a significant challenge for heart surgeons with an extremely high perioperative risk during mitral valve (MV) surgery. The risk is further increased when patients fail to be eligible for any percutaneous treatment, particularly because circumferential calcifications involving the anterior leaflet suggest a critical obstruction of the left ventricular outflow tract (LVOT).

**Objectives:**

The objective of this study was to evaluate residual mitral regurgitation (MR) after surgical mitral valve replacement using a Sapien 3 Ultra (Edwards Lifesciences, CA, USA) transcatheter aortic valve implantation (TAVI) prosthesis, reinforced with a pericardial skirt, in high-risk selected patients with severe MAC.

**Methods:**

Since 2020, five high-risk patients (mean age 70 years; 63–76; four women) with severe mitral disease in the context of severe MAC (computed tomography-based mean MAC Score 8.2 ± 1.1) were operated on after we adopted this novel technique. The operations were performed under general anesthesia, using a transapical TAVI delivery system to position the Sapien 3 in the mitral position under direct vision. To reinforce and avoid paravalvular leakages, a pericardial skirt was previously sewn around the prosthesis, securing it to the annulus and perivalvular atrial surface.

**Results:**

Sapien 3 Ultra implantation was successful without residual MR in all five patients (mild paravalvular leak in one case). Four patients had a 29-mm valve implanted, while one had a 26-mm valve implanted. Predilatation of the native annulus was never performed. Perfusion and clamping times were 134 ± 53 mins and 108 ± 43 mins, respectively. The presence of the pericardial skirt reduced the risk of leakage between the prosthesis and the rigid calcium surface, with final mean and maximal gradients of the TAVI prosthesis of 4.1 and 10.8 mmHg, respectively. There were no left ventricular outflow tract obstructions (mean LVOT gradient of 8 ± 1 mmHg). All patients were discharged, and neither mortality nor prosthetic dysfunction, nor residual mitral regurgitation was recorded. During follow-up, the last patient treated (MAC Score 10, severe calcification of the mitro-aortic junction) returned to our attention with a significant recurrent jet originating from the anterolateral commissure, currently medically treated, given the prohibitive redo risk.

**Conclusion:**

Direct open surgical implantation of the Sapien 3 valve can be safely done in patients with severe MAC in dedicated centers. Reinforcing the TAVI prosthesis by sewing a pericardial skirt led to satisfactory perioperative and early postoperative results, reducing paravalvular leakages. Complex anatomies have a CERTAIN risk of recurrence.

## Introduction

Severe mitral annular calcification (MAC) represents a major challenge for heart surgeons during mitral valve (MV) surgery, usually carrying an extremely high perioperative risk. This risk is further increased when patients fail to be eligible for any percutaneous treatment, particularly because calcifications at the level of the anterior leaflet suggest a critical risk of left ventricular outflow tract obstruction (1). The MAC represents a degenerative process of the mitral annulus, which, in its most characteristic configuration, forms a semilunar deposit of calcium within the posterior annulus, with limited extension to the leaflet tissue. This process differs from the calcifications seen in rheumatic valvular diseases, which usually involve the commissures and the leaflet tissue with only tardive extension to the annulus. Importantly, there are two different MAC presentations, with or without a coexisting MV pathology. The calcifications without valve lesions are usually present in patients with metabolic disorders (e.g., severe diabetes, hyperparathyroidism, and chronic renal failure). They are usually asymptomatic until they develop severe mitral stenosis due to massive annular and sub-annular calcifications.

Conversely, MAC can be present in patients with pre-existing MV disease, particularly in older ones with fibroelastic deficiency or Barlow's or in younger patients with Marfan's syndrome (2, 3). Most commonly, only the posterior annulus is involved, but it may extend to the anterior one or even become massive, involving the full circumference of the mitral valve. The true prevalence of MAC is poorly understood, ranging from 4.3 to 15%, increasing with age and other cardiovascular risk factors, including chronic kidney disease (4, 5).

This report refers to a particular surgical treatment of those rarer, massively calcified forms of MAC. In this setting, complete decalcification can have prohibitive consequences, mandating our institutional policy to surgically implant a Sapien TAVI (Edwards Lifesciences, CA, USA) expandable balloon prosthesis (6–9). Recently, the technique has been optimized by suturing a pericardial skirt around the Sapien 3 prosthesis, radially covering the atrial surface. The rationale aims to rule out any possibility of the paravalvular leaks that boundary this type of strategy. This report aims to identify these patients with severe MAC and to report the results of skirting the Sapien with pericardium to address this massive pathology.

## Methods

### Patient population

From October 2020, five high-risk patients diagnosed with massive, circumferential mitral annular calcification have been operated on in our institution due to MV disease (Table 1). They presented either with an isolated form affecting the MV or in association with concomitant cardiac disease. Each patient was discussed at the heart team meeting. The risk profile was calculated according to the Society of Thoracic Surgeons (STS) Risk Calculator, STS Score. In breif, in preoperative investigations, to prepare patients for mitral surgery and determine the extent of mitral calcification, each patient underwent a coronary angiography examination, transthoracic and transesophageal echocardiography, and a contrast-enhanced MSCT examination in a series. Every patient had the mitral valve calcifications assessed based on multislice computed tomography (MSCT), followed by the Guerrero MAC Score to evaluate their severity (Table 1) (10). The inclusion criteria for this therapy was a surgical candidate who presented with a Guerrero Score > 7 (at least the entire posterior annular calcification involving one or both mitral commissures). Each patient has signed consent to personal data processing for research purposes, and the request for this retrospective study has been sent to the local ethics committee (ID 2022-01188).

**Table 1:** Population baseline.

**Pts #**	**Age**	**Sex**	**STS score**	**Mitral pathology**	**Metabolic disorder**	**Chronic renal failure**	**MAC score (1–10)**	**LVEF preop %**
1	68	Female	2.015%	Degenerative severe MR	Hypothyroidism	No	7	50
2	67	Female	6.771%	Degenerative severe MR	Metabolic syndrome	Yes	9	30
3	76	Female	3.726%	Degenerative severe MR	Hypothyroidism and severe hypertension	No	7	45
4	76	Female	4.357%	Severe MS	Hypothyroidism, diabetes and severe hypertension	No	8	50
5	63	Male	1.466%	Severe MS	Radiation after lymphoma and severe hypertension	No	10	55

### Intraoperative technique

The patient enters the operating room following the institute's standard procedure for MV surgery. Re-evaluation of each patient's cardiac status is carried out by means of transesophageal echocardiography upon intubation. The operation of direct, surgical implantation of a Sapien TAVI prosthesis in the mitral position has already been described by our group (11).

**Figure 1:**
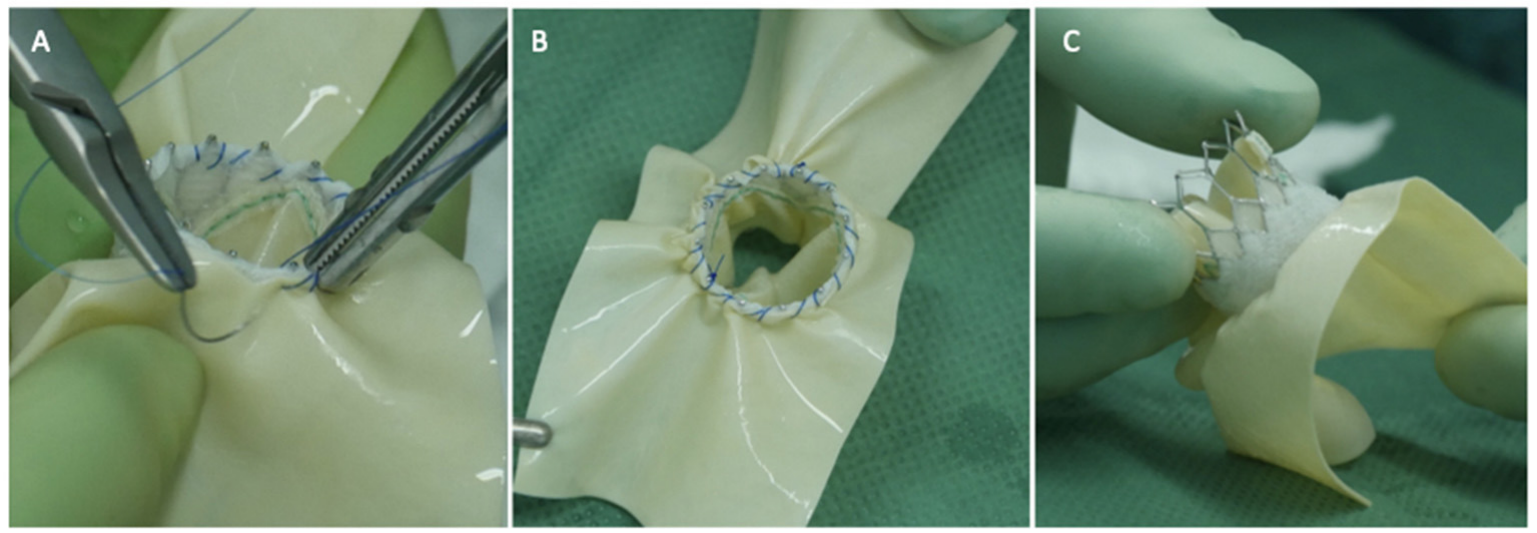
Three panels of figures **(A–C)** depicting the pericardial patch sewn as a skirt around the Sapien 3 Ultra prosthesis (atrial side, the TAVI prosthesis is positioned 180 degrees if compared to an aortic implant).

**Figure 2:**
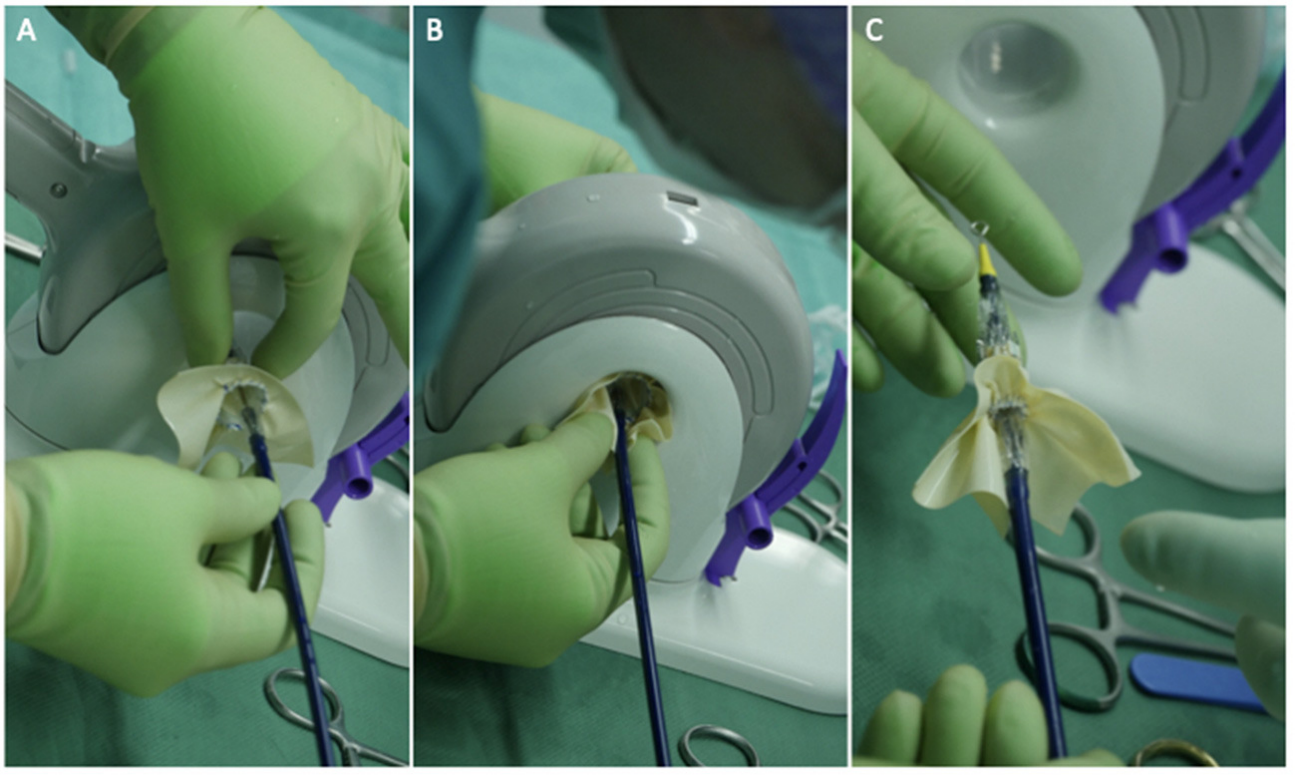
Three panels of figures **(A–C)** depicting the skirted Sapien 3 Ultra when crimped and loaded onto the transapical (shorter) delivery system. The prosthesis should be loaded upside down in relation to the aortic position.

**Figure 3:**
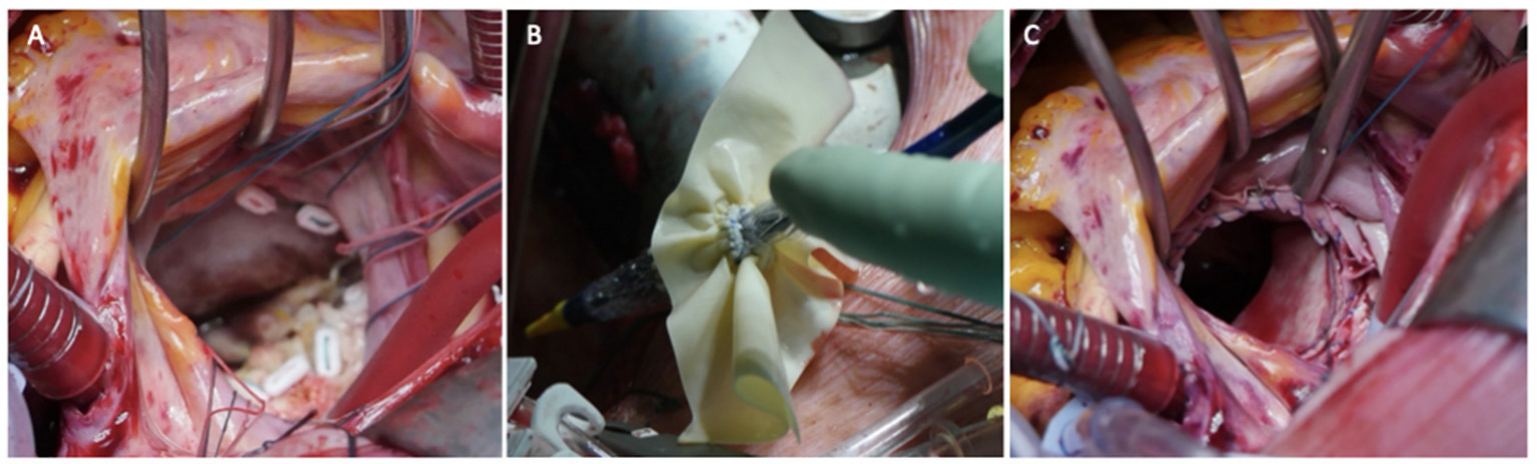
Three panels of figures **(A–C)** depicting the intraoperative placement of pericardial skirt stitches [radial as for a replacement **(A)** the implant **(B)** and the final result **(C)**].

The usual intraoperative strategy continues with median sternotomy, inverted T-opening of the pericardium, and placement of suspension stay-sutures, followed by bicaval cannulation. Once established, the extracorporeal circulation and the venae cavae are both tightened, and the aorta clamped. Del Nido anterograde cardioplegia is administered. With the heart under cardioplegic arrest, the left atrium is linearly incised along Waterston's groove for optimal exposure of the MV. Our policy is to avoid massive debulking in patients with severe calcifications, removing only the anterior mitral leaflet so that the valve can be measured and then replaced. This step is very delicate and must be precise so as not to obstruct the LVOT. It is advisable to flush regularly with a hydro-saline solution to remove any residual calcium. At this time, the size of the Sapien 3 Ultra prosthesis (Edwards Lifesciences, CA, USA) can be measured and confirmed. The pericardial skirt can be sutured on the atrial side (4–0 polypropylene monofilament thread; Figure 1). Here, the recommendation is to have great meticulousness when suturing the Sapien pericardial skirt: it is very easy to have small folds in the sutured pericardium, which becomes moderate leaks under pressure once the circulation has been restarted. Once the valve is ready, it is crimped together with the skirt on a short delivery system (for transapical access TAVI), taking care not to ruin it (Figure 2). To have the correct coaxiality between the MV and the left ventricle, the nose cone was almost completely flexed toward the mitral apex so that the insertion under direct vision becomes physiological. Once accomplished, the pericardium is fixed with other annular pledgeted sutures previously placed through extensive calcifications. The valve is further fixed to the ring with two or four Ethibond 2–0 cardinal stitches (Figure 3).

Once the valve has been properly fixed (by hand-tie or with a Cor-Knot automated fastener as in our case), accurate flushing, closure of the left atrium, and de-airing are performed. Before declamping, we perform a reduced aortotomy to check for residual calcium fragments in the ventricle, ready to embolize.

## Results

The patient population has been described in detail below (Table 1). The TAVI Sapien 3 implantation in the mitral position was successful, with the absence of residual MR in all five patients (mild paravalvular leak in one case). Four patients received a 29-mm valve, while one received a 26-mm valve (Table 2). A predilatation of the native annulus was never performed. Perfusion and clamping times were 134 ± 53 mins and 108 ± 43 mins, respectively. The average time used to sew the pericardial skirt around the Sapien 3 Ultra, after TAVI crimping, was around 7 min. Under transesophageal echocardiographic control, the presence of the pericardial skirt abolished any residual leakage between the prosthesis and the rigid calcium surface, with final mean and maximal gradients of the TAVI prosthesis of 4.1 and 10.8 mmHg, respectively.

**Table 2:** Patient-specific and intraoperative echo details.

**Pts #**	**Age**	**Operation**	**Size (mm)**	**Perfusion Time (mins)**	**Clamping Time (mins)**	**Degree of residual MR**	**Position of residual leak**	**Sapien max/ mean gradient (mmHg)**	**Max LVOT Gradient (mmHg)**	**Baseline vs. Post-op**
**Intraoperative echocardiographical detail**										
1	68	MVR with Sapien 3 Ultra	29	119	97	Absent	No	4 September	8	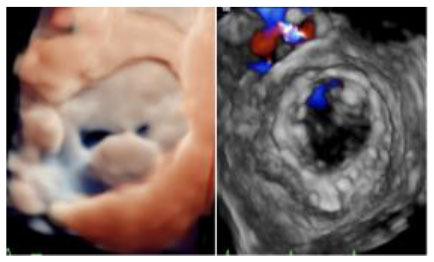
2	67	MVR with Sapien 3 Ultra	29	74	54	Absent	No	5 December	8	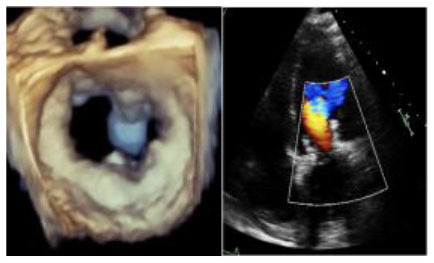
3	76	MVR with Sapien 3 Ultra	29	103	80	Mild	Anterolateral commissure	5 October	10	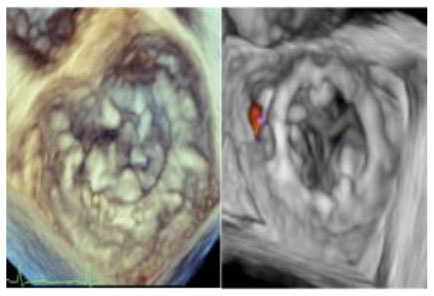
4	76	MVR Sapien 3 Ultra, CABG and LAA occlusion	26	168	138	Absent	No	3 August	9	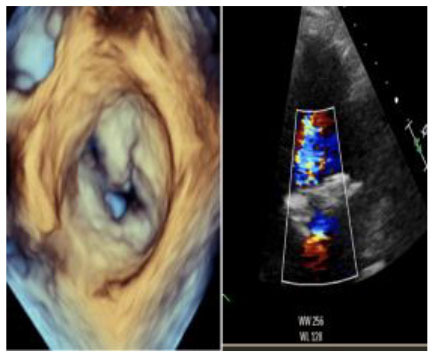
5	63	MVR with Sapien 3 Ultra and bio AVR (Inspiris Resilia 25)	29	208	174	Traces	No	15/5	7	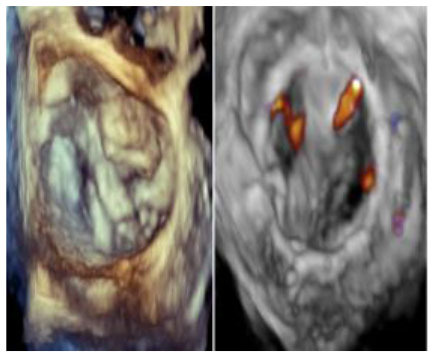

No left ventricular outflow tract obstructions were detected due to the presence or dislocation of the TAVI prosthesis (mean LVOT gradient of 8 ± 1 mmHg). We were able to fix a mild residual leak between the Sapien and the pericardial skirt in two out of five patients using intraoperative transesophageal echocardiographic control (3D zoom with color, “en face” or “surgical” projection of the MV).

There were no deaths, strokes, or major complications during hospitalization. No patient required definitive pacemaker implantation. All patients were discharged, and neither mortality nor prosthetic dysfunction or residual mitral regurgitation was recorded during follow-up. During follow-up, the last patient treated (MAC Score 10, severe calcification of the mitro-aortic junction) returned to our attention with a significant recurrent jet originating from the anterolateral commissure, currently medically treated, given the prohibitive redo risk. The diagnosis was occasionally made during a TEE before electric cardioversion to rule out appendage thrombus. This recurrent mitral jet could not be assessed at TTE, either before or after its recurrence. Most probably, the adherence of the TAVI prosthesis was not complete in the anterolateral commissural region due to massive calcification of the mitroaortic curtain.

### Postoperative care

After surgery, therapeutical oral anticoagulation treatment was adopted and maintained for the first 3 months. If needed, antiarrhythmic drugs (AADs) were continued during the first 3–6 months. All patients visited the outpatient clinic according to the standard protocol of care at our institution.

## Discussion

We report on the validity of reinforcing the Sapien 3 TAVI prosthesis with a pericardial skirt when implanted in mitral valves with severe MAC. Taking into account the complexity of the population treated, the intraoperative and short-term outcomes achieved with this technique thus far are very promising (Table 1). Notwithstanding, a certain risk of recurrence cannot be excluded, as there has been a recurrence in the last treated patient (MAC Score 10). Most probably, the adherence of the TAVI prosthesis was not complete in the anterolateral commissural region due to massive calcification of the mitroaortic curtain.

In our opinion, the most important issue related to this technique is patient selection. Theoretically, the Sapien 3 in MAC works well in every patient and has no absolute contraindications, contrary to transcatheter MV implantation (TMVI), which can have limitations due to LVOT obstruction. At our institute, the policy is to deliver the aforementioned therapy only in the case of severe MAC. With this approach, even all kinds of MAC become treatable. According to the Guerrero Score, which was created to objectively assess the severity of native mitral valve calcifications based on MSCT, our patients were severely calcific (10). We could not calculate the final MV area and plan the prosthesis size, as the anterior leaflet would be removed to a variable extent. Therefore, it is not feasible to predict the size of the Sapien 3, which will be implanted.

The advantage of surgical implantation of Sapien in MAC is the removal of the anterior leaflet. This eliminates the risk of LVOT obstruction, which is not reachable with TMVI prostheses in these cases of circumferential MAC. The outflow tract obstruction is currently the Achilles heel of TMVI, even more relevant for MAC implants (1, 12, 13).

The postoperative LVOT gradients of our patients never exceeded 12–15 mmHg (Table 2). Surgical removal of the anterior calcific leaflet also allows greater safety in removing calcific fragments. In this extreme MAC setting, the Lampoon technique cannot be performed, as the anterior leaflet is severely calcified and non-approachable (14).

The experience with Sapien in patients with severe MAC has grown over the years at our institution: initially rudimentary use of the Sapien XT, followed by the Sapien 3 with extra annular fixations, and currently the pericardial reinforced Sapien 3 Ultra (6, 11, 15). Indeed, the pericardial skirt around the Sapien prosthesis radially covers the atrial surface around the mitral annulus. “Skirting” the atrium with radial stitches prevents leaks between the native contour of the mitral ring and the prosthesis, which, being circular and not oval, may not be sealed. The pericardial skirt's effect in abolishing leaks is to create a low-flow chamber consisting of the atrial wall, the pericardium skirt sutured around the prosthesis, and the calcific ring. Even if the pericardial structure goes toward a certain degree of calcification around the Sapien without interfering with the prosthetic leaflets, it should not necessarily become a problem because it would reduce the onset of PV leaks.

In comparison to the past, the improvement is evident. Without the skirt, the whole residual regurgitation was paravalvular between the inextensible calcific ring and the TAVI Sapien 3 prosthesis. Adding the pericardial skirt significantly reduced residual leakages between the prosthesis and the rigid calcium surface. In our opinion, this technique offers some strengths that deserve further investigation.

### Solutions to address MAC

The surgical strategies for a calcified mitral ring should usually be adapted to the extent of the calcifications and the surgeon's experience level. Hence, the decalcification en bloc of the posterior mitral ring and its reconstruction should be the strategy of choice when the calcification is limited to the posterior leaflet or slightly involving the anterior. Decalcification becomes problematic when the extent increases and fully involves both mitral leaflets and the trigones; hence, a “conservative” approach is more convenient. According to European and American guidelines, surgery represents the treatment of choice for MV disease (16, 17). However, several patients were deemed unsuitable for surgery due to a prohibitive/high operative risk. Today, TMVI has grown as a possible alternative to surgery. Current data about TMVI are still limited and come from different settings: valve-in-native MV, valve-in-valve (ViV), valve-in-ring (ViR), and valve-in-mitral annular calcification, our field of interest. There are many promising devices for TMVI. However, they are all in the very early stages of evaluation. Their role in severe MAC may be limited as these self-expandable devices are specifically designed for non-calcified pathology and may not have the radial strength needed to treat massively calcified stenotic mitral valves. The outcomes of TMVI for patients with degenerated bioprosthesis failed annuloplasty rings, and severe MACs have been recently reported in a multicenter registry by Yoon et al. involving 521 patients (1). Indeed, the most complex scenario was represented by ViMAC, with a technical success of 62%, while 30-day mortality and 1-year mortality were 34.5 and 62.8%, respectively. In addition to this registry, the data obtained from the STS/Transcatheter Valve Therapies (STS/TVT) registry confirmed the same trend for the three groups (9). Of note, in the STS/TVT registry, the occurrence of LVOTO in the ViMAC group was much lower (10%) than Yoon's above-mentioned experience (39.7%). The latter might be explained by the non-univocal definition used for LVOTO among these registries.

### Reflections

The conservative approach (removal of the anterior leaflet only without annular decalcification) may present some limitations compared to more radical techniques, but it is profitable in terms of speed (less than an hour for a mitral replacement in a very complex setting, Table 2). The risks of rupturing the atrioventricular groove can be avoided by avoiding decalcification and patch reconstruction techniques. Another important issue is the presence of a biological TAVI prosthesis in the mitral position. The durability of standard surgical bioprostheses is reduced compared to the aortic position, and the altered calcium metabolism of these patients and the high prevalence of severe renal dysfunction are not favorable prognostic factors. Durability is poorly known in the mitral position and is unpredictable. Last, the role of therapeutic anticoagulation after the third month is unclear. Its interruption is advised for stented surgical bioprosthesis but is unclear in this setting.

Last, from a patient-prosthesis mismatch perspective, once the anterior leaflet was removed, the smallest of the stented surgical bioprostheses available did not pass through the calcific annulus in two cases. If the intention is not to touch these rocky annuli, a valuable alternative is to implant a stentless TAVI prosthesis with excellent gradients (Table 2).

## Conclusion

Direct open surgical implantation of the Sapien 3 valve can be safely carried out in patients with massive mitral calcifications in dedicated institutions. Reinforcing the TAVI prosthesis by sewing a pericardial skirt around it, fixed to the annulus and the perivalvular atrial surface, led to very satisfactory perioperative and early postoperative results, abolishing paravalvular leakages. Further, the removal of the anterior leaflet eliminates the outflow tract obstruction. Proper patient selection, namely massive MACs, is crucial to maximizing the benefits of this hybrid treatment.

## Data availability statement

The raw data supporting the conclusions of this article will be made available by the authors, without undue reservation.

## Ethics statement

The studies involving human participants were reviewed and approved by the Comitato Etico Canton Ticino (CH) – ID 2022-01188. The patients/participants provided their written informed consent to participate in this study.

## Author contributions

AP and TT substantial contributions to the acquisition and analysis of data for the work. AP drafted the work. AP and EF substantial contributions to the design of the work. GP, SD, and EF revised it critically for important intellectual content and provided approval for publication of the content. All authors contributed to the article and approved the submitted version.

## Funding

This study was supported by the Institutional funding.

## Conflict of interest

Author EF is a consultant for Edwards Lifesciences. The remaining authors declare that the research was conducted in the absence of any commercial or financial relationships that could be construed as a potential conflict of interest.

## Publisher's note

All claims expressed in this article are solely those of the authors and do not necessarily represent those of their affiliated organizations, or those of the publisher, the editors and the reviewers. Any product that may be evaluated in this article, or claim that may be made by its manufacturer, is not guaranteed or endorsed by the publisher.

## References

[1] Yoon SH Whisenant BK Bleiziffer S Delgado V Dhoble A Schofer N. Outcomes of transcatheter mitral valve replacement for degenerated bioprostheses, failed annuloplasty rings, and mitral annular calcification. Eur Heart J. (2019) 40:441–51. 10.1093/eurheartj/ehy590 30357365

[2] Savage DD Garrison RJ Castelli WP McNamara PM Anderson SJ Kannel WB. Prevalence of submitral (anular) calcium and its correlates in a general population-based sample (the Framingham Study). Am J Cardiol. (1983) 51:1375–8. 10.1016/0002-9149(83)90315-6 6846165

[3] Carpentier AF Pellerin M Fuzellier JF Relland JY. Extensive calcification of the mitral valve anulus: pathology and surgical management. J Thorac Cardiovasc Surg. (1996) 111:718–29. 10.1016/S0022-5223(96)70332-X 8614132

[4] Fox CS Vasan RS Parise H Levy D O'Donnell CJ D'Agostino RB. Framingham Heart Study. Mitral annular calcification predicts cardiovascular morbidity and mortality: the Framingham heart study. Circulation. (2003) 107:1492–6. 10.1161/01.CIR.0000058168.26163.BC 12654605

[5] Elmariah S Budoff MJ Delaney JA. Risk factors associated with the incidence and progression of mitral annulus calcification: the multi-ethnic study of atherosclerosis. Am Heart J. (2013) 166:904–12. 10.1016/j.ahj.2013.08.015 24176447 PMC3978772

[6] Ferrari E Dvir D Guerrero M. Transcatheter mitral valve replacement in degenerated calcified native mitral valves: is the currently available technology suitable? Eur J Cardiothorac Surg. (2016) 50:391–5. 10.1093/ejcts/ezw188 27247377

[7] Guerrero M Dvir D Himbert D Urena M Eleid M Wang DD. Transcatheter mitral valve replacement in native mitral valve disease with severe mitral annular calcification: results from the first multicenter global registry. JACC Cardiovasc Interv. (2016) 9:1361–71. 10.1016/j.jcin.2016.04.022 27388824

[8] Guerrero M Urena M Himbert D Wang DD Eleid M Kodali S. 1-year outcomes of transcatheter mitral valve replacement in patients with severe mitral annular calcification. J Am Coll Cardiol. (2018) 71:1841–53. 10.1016/j.jacc.2018.02.054 29699609

[9] Guerrero M Vemulapalli S Xiang Q Wang DD Eleid M Cabalka AK. Thirty-day outcomes of transcatheter mitral valve replacement for degenerated mitral bioprostheses (valve-in-valve), failed surgical rings (valve-in-ring), and native valve with severe mitral annular calcification (valve-in-mitral annular calcification) in the united states: data from the society of thoracic surgeons/american college of cardiology/transcatheter valve therapy registry. Circ Cardiovasc Interv. (2020) 13:e008425. 10.1161/CIRCINTERVENTIONS.119.008425 32138529

[10] Guerrero M Wang DD Pursnani A Eleid M Khalique O Urena M. Cardiac computed tomography-based score to categorize mitral annular calcification severity and predict valve embolization. JACC Cardiovasc Imaging. (2020) 13:1945–57. 10.1016/j.jcmg.2020.03.013 32417332

[11] Gallo M Demertzis S Torre T Ferrari E. Direct surgical transcatheter heart valve implantation in a calcified mitral valve. Multimed Man Cardiothorac Surg (2018). 10.1510/mmcts.2018.053 30285323

[12] Russo G Maisano F Massaro G Terlizzese G Mariano E Bonanni M. Challenges and Open Issues in Transcatheter Mitral Valve Implantation: Smooth Seas Do Not Make Skillful Sailors. Front Cardiovasc Med. (2022) 8:738756. 10.3389/fcvm.2021.738756 35224022 PMC8863742

[13] Sorajja P Gössl M Babaliaros V Rizik D Conradi L Bae R. Novel transcatheter mitral valve prosthesis for patients with severe mitral annular calcification. J Am Coll Cardiol. (2019) 74:1431–40. 10.1016/j.jacc.2019.07.069 31514943

[14] Khan JM Babaliaros VC Greenbaum AB Foerst JR Yazdani S McCabe JM. Anterior leaflet laceration to prevent ventricular outflow tract obstruction during transcatheter mitral valve replacement. J Am Coll Cardiol. (2019) 73:2521–34. 10.1016/j.jacc.2019.02.076 31118146 PMC6664295

[15] Ferrari E Niclauss L Locca D Marcucci C. On-pump fibrillating heart mitral valve replacement with the SAPIEN™ XT transcatheter heart valve. Eur J Cardiothorac Surg. (2014) 45:749–51. 10.1093/ejcts/ezt364 23847181

[16] Vahanian A Beyersdorf F Praz F Milojevic M Baldus S Bauersachs J. 2021 ESC/EACTS Guidelines for the management of valvular heart disease: Developed by the Task Force for the management of valvular heart disease of the European Society of Cardiology (ESC) and the European Association for Cardio-Thoracic Surgery (EACTS). Eur Heart J. (2022) 43:561–632. 10.1093/eurheartj/ehab395 34453165

[17] Otto CM Nishimura RA Bonow RO Carabello BA Erwin JP Gentile F. 2020 ACC/AHA Guideline for the management of patients with valvular heart disease: executive summary: a report of the american college of cardiology/american heart association joint committee on clinical practice guidelines. Circulation. (2021) 143:e35–71. 10.1161/CIR.0000000000000932 33332149

